# Research on permanent magnet synchronous motor algorithm based on linear nonlinear switching self-disturbance rejection control

**DOI:** 10.1038/s41598-023-46881-8

**Published:** 2023-11-16

**Authors:** Xiangde Liu, Yu Li, Liang Xia, Xianfeng Tan, Xiang Cao

**Affiliations:** 1 Chongqing University of Posts and Telecommunications, Chongqing, 400065 China; 2 Chongqing Robotics Institute, Chongqing, 400000 China

**Keywords:** Engineering, Electrical and electronic engineering

## Abstract

This paper presents a linear-nonlinear switching control strategy, called Switching Active Disturbance Rejection Control (SADRC), to enhance the disturbance rejection capability of the speed controller in a servo system. SADRC combines the advantages of Linear Active Disturbance Rejection Control (LADRC) and Nonlinear Active Disturbance Rejection Control (NLADRC), and introduces a parameter to switch between nonlinear and linear control, thereby improving the robustness of the servo system. Firstly, the mathematical model of the motor is analyzed as the starting point of the paper. Then, the basic principles of Active Disturbance Rejection Control (ADRC) are analyzed, and improvements are made to address its limitations, resulting in the design of SADRC. The parameters introduced in SADRC are analyzed to determine their appropriate ranges. Finally, the performance of SADRC is validated by comparing the rotational effects of Permanent Magnet Synchronous Motor (PMSM).

## Introduction

It is well known that the servo system takes the difference between the set value and the feedback value as input. The servo system is commonly used in fields such as industrial robotic arms, aerospace, and CNC machine tools, which require high motor performance and control accuracy^1, 2^. Taking industrial robotic arms as an example, the control performance of the servo system directly determines the production speed and product quality of the production line. Therefore, improving the robustness of the servo system is of great significance. The overall performance of the servo system is mainly determined by two parts. One is the hardware aspect, such as the operating speed and control frequency of the control chip, the accuracy of the encoder, the motor structure^3–5^, and the motor type^6^. The other aspect is the control algorithm, and different control algorithms also determine the performance of the entire system. Among them, the speed controller and current controller commonly use PI control. Due to its simplicity and good performance, PI control is widely used in the industrial control field. However, with the continuous improvement of industrial requirements, ordinary PI control is difficult to meet the requirements of high-performance servo systems. It is of great significance to improve the robustness of the servo system through optimized control algorithms. Many scholars have conducted in-depth research on control algorithms, such as proportional-integral (PI) control with feedforward compensation, disturbance-based control^7^, Sliding Mode Control (SMC)^8–10^, Model Predictive Direct Speed Control (MP-DSC) ^11^ and Active Disturbance Rejection Control (ADRC), etc.

ADRC was initially proposed by researcher Han^12^. It is a nonlinear structure created by combining modern control theory with the PID controller. Subsequently, through the efforts of scholars such as Gao Zhiqiang, the bandwidth method was used to solve the parameter tuning and linearization issues of ADRC, enabling its application in engineering practice^13, 14^. However, Linear Active Disturbance Rejection Control (LADRC) has a constant gain, which leads to large initial state errors and delayed response speed. In comparison, Nonlinear Active Disturbance Rejection Control (NLADRC) has higher tracking accuracy, stronger disturbance rejection capability, and faster response. However, the stability and reliability analysis of NLADRC is extremely difficult, which greatly hinders the practical application of ADRC^15, 16^. Therefore, how to further enhance the robustness of the ADRC controller remains a challenge. In this regard, some scholars have conducted further research based on the ADRC framework. For example, Li et al. used the ADRC algorithm with an Extended State Observer (ESO) to suppress disturbances in control systems^17^. Qi provided proof of stability for NLADRC^18, 19^. Zhao demonstrated the convergence of nonlinear active disturbance rejection, providing theoretical support for its application^20^. On the other hand, combining the advantages of LADRC and NLADRC can greatly improve system performance. Hao directly selected LADRC and NLADRC based on the magnitude of the system input error. Although this approach is simple to operate, it involves complex calculations, especially during controller switching moments^21^. Lin designed the ESO module, but the constructed function had discontinuous states^22^. In this paper, we construct the ESO module, add parameters, and achieve a smooth transition from nonlinear to linear by designing a Switching Active Disturbance Rejection Control (SADRC) that combines the advantages of LADRC and NLADRC. Finally, the performance of SADRC is validated through comparative experimental data on Permanent Magnet Synchronous Motor (PMSM).

## Mathematical model of permanent magnet synchronous motor

The object of control in this study is the permanent magnet synchronous motor. Assuming that the windings are symmetrical and disregarding core saturation, eddy current loss, and hysteresis loss, the mathematical model of a PMSM can be derived based on motor control theory. The stator-side voltage equation is shown in the following Eq. (1).

1 $$\left\{\begin{array}{c}{u}_{d}={R}_{s}{i}_{d}+\frac{d{\psi }_{d}}{dt}-\omega {\psi }_{q}\\ {u}_{q}={R}_{s}{i}_{q}+\frac{d{\psi }_{q}}{dt}-\omega {\psi }_{d}\end{array}\right.$$

where ${u}_{d}$, ${u}_{q}$ is the stator voltage component of the d-q coordinate system, ${\psi }_{d}$ ${\psi }_{q}$ is the stator flux component, and $\omega$ is the rotational angular velocity. The flux equation is (2).

2 $$\left\{\begin{array}{c}{\psi }_{q}={L}_{q}{i}_{q}\\ {\psi }_{d}={L}_{d}{i}_{d}+{\psi }_{f}\end{array}\right.$$

where ${L}_{d}$, ${L}_{q}$ is the inductance component of the d and q axes, and ${\psi }_{f}$ is the permanent magnet flux linkage.

This can be obtained from the above formula:

3 $$\left\{\begin{array}{c}{u}_{d}={R}_{s}{i}_{d}+{L}_{d}\frac{d{i}_{d}}{dt}-{\omega }_{r}{L}_{qq}{i}_{q}\\ {u}_{q}={R}_{s}{i}_{q}+{L}_{q}\frac{d{i}_{q}}{dt}+{\omega }_{r}{L}_{d}{i}_{d}+{\omega }_{r}{\psi }_{f}\end{array}\right.$$

Electromagnetic torque equation for PMSM:

4 $${T}_{e}=\frac{3}{2}{P}_{n}\left[{\psi }_{f}{i}_{q}+\left({L}_{d}-{L}_{q}\right){i}_{d}{i}_{q}\right]$$

Among them ${T}_{e}$ is electromagnetic torque; ${P}_{n}$ is the number of pole pairs, and other parameters are the same as above. Mechanical equations of motion for PMSM:

5 $${T}_{e}-{T}_{l}=J\frac{d{w}_{r}}{dt}$$

where ${T}_{l}$ is the load torque; $J$ is the moment of inertia.

## ADRC control principle

### LADRC control principle

ADRC is mainly composed of three parts: the tracking differentiator, the extended state observer, and the state error feedback control rate. The core idea of self-disturbance rejection control is to proactively extract disturbance information from the input/output signal of the controlled object before it significantly affects the final output of the system. This information is then used to eliminate the disturbance as quickly as possible using the control signal, thereby minimizing its impact on the controlled quantity.

The control object in this paper is a first-order system, specifically a permanent magnet synchronous motor. This type of motor is primarily utilized in the field of industrial robotic arms. The following state-space equation can be obtained. This is shown in the following Eq. (6).

6 $$\left\{\begin{array}{c}\dot{{x}_{1}}={x}_{2}+bu\\ \dot{{x}_{2}}=\dot{f\left({x}_{1},{x}_{2}\right)}\end{array}\right.$$

The lower ${x}_{1}$ is the system variable, $f\left({x}_{1},{x}_{2}\right)$ is the total disturbance of the system, $b$ is the estimate of the gain of the control system, and $u$ is the system input variable. The permanent magnet synchronous motor adopts a double closed-loop vector control system. The current loop utilizes traditional PI control, while the speed loop utilizes the SADRC. The control system is an inertial system. It takes a certain amount of time for the speed to reach the desired speed from zero. At the initial moment, there is a significant difference between the desired speed and the velocity feedback, resulting in a large overshoot. To achieve this, the transition process is designed to gradually increase the velocity. The equation for this process is as follows:

7 $$\left\{\begin{array}{l}{\dot{v}}_{1}={v}_{2}\\ {\dot{v}}_{2}=-{r}^{2}\left({v}_{1}-{v}_{0}\left(t\right)\right)-2r{v}_{2}\end{array}\right.$$

${v}_{0}\left(t\right)$ represents the given speed and $r$ is the speed factor. ${v}_{1}$ is the tracking value of ${v}_{0}\left(t\right)$, ${v}_{2}$ is the derivative of ${v}_{1}$ and $r$ is the speed factor. ESO is a vital part of ADRC for real-time estimation of system variables, real-time estimation and compensation of total disturbances, elimination of disturbances, and improved control. For first-order systems, LESO can be designed as follows:

8 $$\left\{\begin{array}{l}e={z}_{1}-{v}_{f}\\ {\dot{z}}_{1}={z}_{2}-{\beta }_{1}{{^{\prime}}}e+{b}_{0}u\\ {\dot{z}}_{2}=-{\beta }_{2}{{^{\prime}}} \, e\end{array}\right.$$

where ${z}_{1}$, ${z}_{2}$ are the state variables of the observer, ${z}_{1}$ are used to track ${v}_{1}$, ${z}_{2}$ is the estimation of the total perturbation, ${\beta }_{1}{{^{\prime}}}$, ${\beta }_{2}{{^{\prime}}}$ are the gain factors of ${z}_{1}$, and ${z}_{2}$, respectively, and *e* is the error between the estimated value and the output value. The output of LESO is as follows:

9 $$u=\frac{{u}_{0}-{z}_{2}}{{b}_{0}}$$

In a first-order linear system, LSEF can design P control:

10 $${u}_{0}={K}_{1}\left({v}_{1}-{z}_{1}\right)$$

where ${K}_{1}$ are the gain factors of the error.

### NLADRC control principle

The first-order NLADRC transition process is improved on LADRC:

11 $$\left\{\begin{array}{c}{\dot{v}}_{1}={v}_{2}\\ {\dot{v}}_{2}= \, {\text{f}}{\text{h}}{\text{a}}{\text{n}} \, \left({v}_{1}-{v}_{0}(t),{v}_{2},r,h\right)\end{array}\right.$$

$h$ is the step size. "${\text{fhan}}$" is the fastest tracking function, as follows:

12 $$\left\{\begin{array}{l}d=r{h}^{2},{a}_{0}=h{x}_{2},y={x}_{1}+{a}_{0},{a}_{1}=\sqrt{d\left(d+8\left|y\right|\right)}\\ {a}_{2}={a}_{0}+sign(y)\left({a}_{1}-d\right)/2\\ {a}_{3}=(sign(y+d)-sign(y-d))/2\\ {a}_{4}=\left({a}_{0}+y-{a}_{2}\right){a}_{3}+{a}_{2}\\ {a}_{5}=\left(\mathit{sign}\left({a}_{4}+d\right)-\mathit{sign}\left({a}_{4}-d\right)\right)/2\\ \, {\text{f}}{\text{h}}{\text{a}}{\text{n}} \, \left({x}_{1},{x}_{2},r,h\right)=-r\left(\frac{{a}_{4}}{d}-\mathit{sign}\left({a}_{4}\right)\right){a}_{5}-rsign\left({a}_{4}\right)\end{array}\right.$$

The transition process of NLADRC and LADRC is similar, and f can be replaced with the "${\text{fhan}}$" function. NLESO introduces the nonlinear function $fal\left(e,{\alpha }_{i},\delta \right)$, which makes the observer have the characteristics of "small error, large gain, large error, small gain", the specific expression is as follows:

13 $$\left\{\begin{array}{l}e={z}_{1}-y\\ {\dot{z}}_{1}={z}_{2}-{\beta }_{1}fal\left(e,{\alpha }_{1},\delta \right)+{b}_{0}u\\ {\dot{z}}_{2}=-{\beta }_{2} \, fal\left(e,{\alpha }_{1},\delta \right)\end{array}\right.$$

where the expression for the nonlinear function $fal\left(e,{\alpha }_{i},\delta \right)$ is:

14 $$fal\left(e,{\alpha }_{i},\delta \right)=\left\{\begin{array}{l}\frac{e}{{\delta }^{1-{\alpha }_{i}}}\,\,\,\,\,\,\,\,\,\,\,\,\,\,\,\,\,\,\,\,\,|e|\le \delta \\ |e{|}^{{\alpha }_{i}}sign(e)\,\,\,\,|e|>\delta \end{array}\right.$$

where ${\beta }_{1}$, ${\beta }_{2}$ is the gain factor, ${\alpha }_{1}$, ${\alpha }_{2}$, and $\delta$ are pending parameters. The nonlinear state error feedback rate is also introduced into the nonlinear $fal\left(e,{\alpha }_{i},\delta \right)$ function as follows:

15 $${u}_{0}={k}_{1}\text{ fal }\left({v}_{1}-{z}_{1},{\alpha }_{1}{{^{\prime}}},{\delta }{{^{\prime}}}\right)$$

Among them, ${k}_{1}$ is tunable parameters, and ${\alpha }_{1}{{^{\prime}}}$ and ${\delta }{{^{\prime}}}$ are the two pending parameters of NLSEF.

## SADRC design

**Figure 1:**
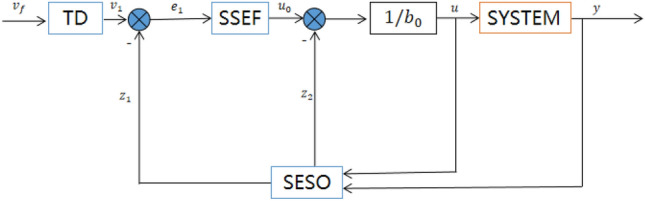
SADRC flow block diagram.

LADRC and NLADRC have their own advantages. LADRC is characterized by easy parameter adjustment and easy implementation in engineering. It is particularly effective when the error is large, as it allows for a large control gain. NLADRC has the characteristics of "small error, large gain, large error, small gain". In other words, NLADRC has a stronger adjustment ability when the error is small. As a switch, SADRC switches to NLADRC when the error is small and LADRC when the error is large, effectively utilizing the benefits of both LADRC and NLADRC. The framework of SADRC is shown in Fig. 1, ${v}_{f}$ the speed is given, and the speed step increases uniformly in the TD part. SESO is a system expansion state observer used to detect system state variables in real time. SSEF is the state error feedback rate, the input error signal is processed, the output ${u}_{0}$ is different from the compensation value of the observer. Finally, it is input into the control system.

In the TD section, there is no clear distinction between LADRC and NLADRC, and there is no need to create a separate toggle switch. In the experiment, the discrete form of the TD part is designed as follows.

16 $$\left\{\begin{array}{l}f=-{r}^{2}\left({v}_{1}-{v}_{f}\right)-2r{v}_{2}\\ {v}_{1}={v}_{1}+{v}_{2}h\\ {v}_{2}={v}_{2}+fh\end{array}\right.$$

where $f$ and ${v}_{2}$ are intermediate variables, and ${v}_{f}$ is given velocity.

### Design of SESO and SSEF

**Figure 2:**
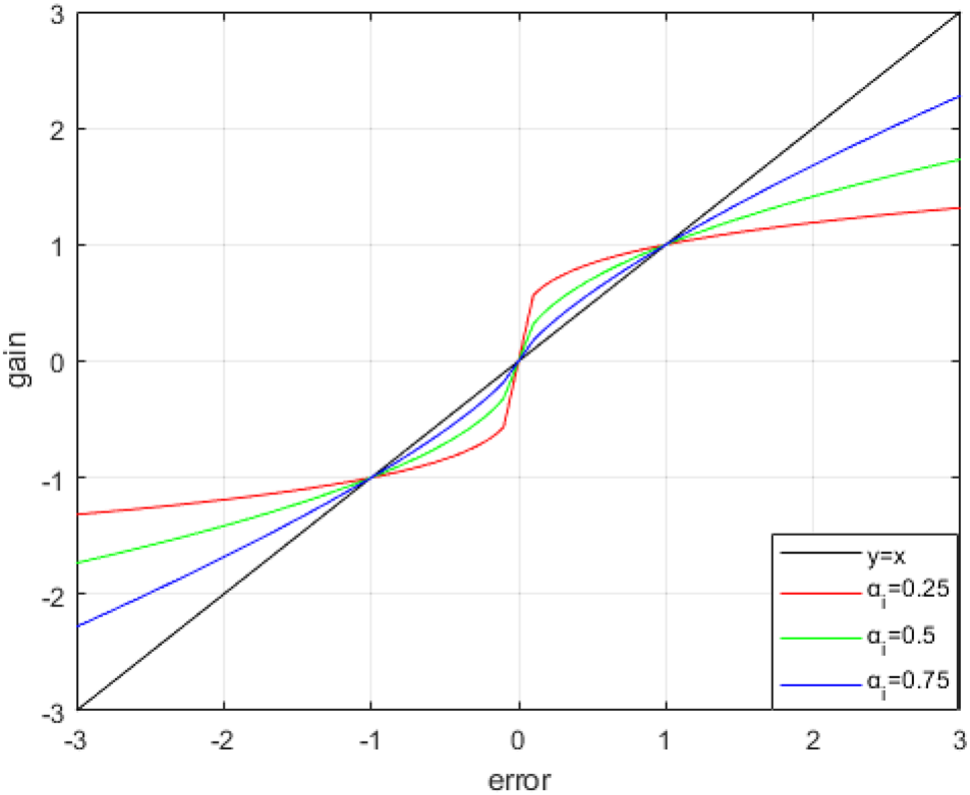
$$fal\left(e,{\alpha }_{i},\delta \right)$$ function curve.

The core of SADRC lies in the design of the $fa{l}_{s}\left(e,{\alpha }_{i},{\delta }_{1},{\delta }_{2}\right)$ function in SESO, which not only needs to meet the good control effect of NLADRC in the case of "small error", but also requires LADRC to have a large gain when the error is large. Figure 2 is the function curve of $fal\left(e,{\alpha }_{i},\delta \right)$.

It can be seen from the curve that the smaller the value of the ${\alpha }_{i}$, the stronger the nonlinearity of the $fal\left(e,{\alpha }_{i},\delta \right)$ function. Additionally, there is a greater the gain in the case of small errors, but the increase in gain is slower for large errors. In addition, δ is the critical point of the linear interval of $fal\left(e,{\alpha }_{i},\delta \right)$, the smaller the *δ*, the smaller the linear interval of the $fal\left(e,{\alpha }_{i},\delta \right)$ function, the stronger the nonlinearity. In NLADRC, generally through continuous debugging ${\alpha }_{i}$, $\delta$ two parameters make the control effect of the system moderate, but it is impossible to ensure that the system can maintain a high control effect when the error changes in a large range. Therefore, this paper designs a class of function curves that maintain high gain and fast response speed even if the error fluctuates in a wide range, and the $fa{l}_{s}\left(e,{\alpha }_{i},{\delta }_{1},{\delta }_{2}\right)$ function is designed as follows:

17 $$fa{l}_{s}\left(e,{\alpha }_{i},{\delta }_{1},{\delta }_{2}\right)=\left\{\begin{array}{l}{e\delta }_{1}^{{\alpha }_{i}-1}\,\,|e|\le {\delta }_{1}\\ {\left|e\right|}^{{\alpha }_{i}}sign(e)\,\,\,\,{\delta }_{1}<|e|<{\delta }_{2}\\ {K}_{c}e{\delta }_{2}^{{\alpha }_{i}-1}\,\,\,\,|e|\ge {\delta }_{2}\end{array}\right.$$

**Figure 3:**
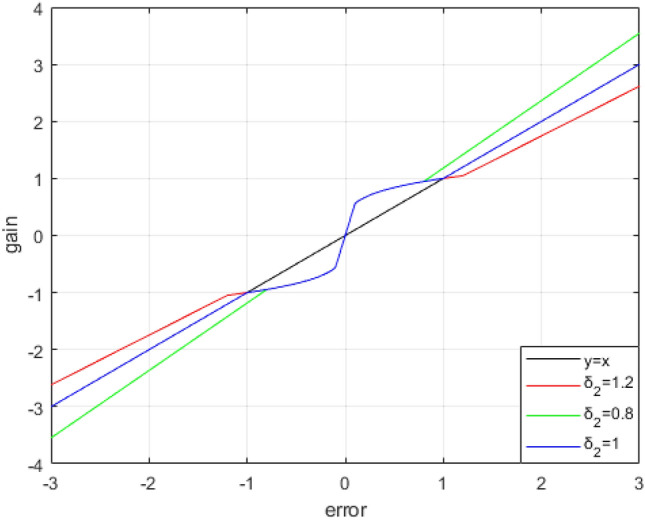
$$fa{l}_{s}\left(e,{\alpha }_{i},{\delta }_{1},{\delta }_{2}\right)$$ curves.

Among them, in order to ensure the system performance, the ${K}_{c}$ generally takes a value of 1, which can be adjusted according to the needs of the system. $fa{l}_{s}\left(e,{\alpha }_{i},{\delta }_{1},{\delta }_{2}\right)$ function compared with the $fal\left(e,{\alpha }_{i},\delta \right)$ function, the introduction of ${\delta }_{2}$, ${\delta }_{2}$ changes the gain of the controller under large error conditions, that is, the advantages of LADRC in large error conditions are fused. An image of the $fa{l}_{s}\left(e,{\alpha }_{i},{\delta }_{1},{\delta }_{2}\right)$ function is shown below. It can be seen from the curve that when the error range is $-{\delta }_{1}<e<{\delta }_{1}$, SADRC presents nonlinear characteristics, when the error range is $|e|\ge {\delta }_{2}$, SADRC has the properties of LADRC, $fa{l}_{s}\left(e,{\alpha }_{i},{\delta }_{1},{\delta }_{2}\right)$ function combines the advantages of LADRC and NLADRC. The effect of ${\delta }_{2}$ on the system is shown in Fig. 3.

In this experiment, SADRC is applied to the servo-controlled speed loop, so the system output *y* is the speed feedback ${v}_{f}$, then SESO can be designed:

18 $$\left\{\begin{array}{l}e={z}_{1}-{v}_{f}\\ {\dot{z}}_{1}={z}_{2}-{\beta }_{1}fa{l}_{s}\left(e,{\alpha }_{1},{\delta }_{1},{\delta }_{2}\right)+{b}_{0}u\\ {\dot{z}}_{2}=-{\beta }_{2} \, fa{l}_{s}\left(e,{\alpha }_{2},{\delta }_{1},{\delta }_{2}\right)\end{array}\right.$$

where ${z}_{1}$ is the state variable given by the tracking speed, ${z}_{2}$ is the system interference value, ${\beta }_{1}$, ${\beta }_{2}$ are the coefficients to be determined, and u is the system output, as follows:

19 $$u=\frac{{u}_{0}-{z}_{2}}{{b}_{0}}$$

${u}_{0}$ is the output of SSEF, this experiment uses first-order SADRC, so SSEF directly uses proportional control, and SSEF is designed as:

20 $${u}_{0}={k}_{p} \, fa{l}_{s}\left(e,{\alpha }_{1}{{^{\prime}}},{\delta }_{1}{{^{\prime}}},{\delta }_{2}{{^{\prime}}}\right)$$

### SADRC parameter tuning

#### SESO

The extended state observer is the core of the disturbance rejection control, which observes and compensates for the system disturbance in real time. The parameters to be determined are: ${\beta }_{1}$, ${\beta }_{2}$, ${\alpha }_{1}$, ${\alpha }_{2}$, ${\delta }_{1}$, ${\delta }_{2}$. ${\beta }_{1}$, ${\beta }_{2}$ can be adjusted according to the bandwidth method. In order to ensure system stability the range of ${\alpha }_{1}$, ${\alpha }_{2}$, ${\delta }_{1}$, ${\delta }_{2}$ can be reduced to: ${0<\alpha }_{1}<{\alpha }_{2}<1$, ${0<\delta }_{1}{<\delta }_{2}\le 1$, the specific parameter setting process is as follows.

(18) is obtained by performing the Laplace transform:

21 $$\left\{\begin{array}{l}e={z}_{1}-{v}_{f}\\ {\mathrm{sz}}_{1}={z}_{2}-{\beta }_{1}fa{l}_{s}\left(e,{\alpha }_{1},{\delta }_{1},{\delta }_{2}\right)+{b}_{0}u\\ {\mathrm{sz}}_{2}=-{\beta }_{2} \, fa{l}_{s}\left(e,{\alpha }_{2},{\delta }_{1},{\delta }_{2}\right)\end{array}\right.$$

The bandwidth method for parameter tuning reference, let $fa{l}_{s}\left(e,{\alpha }_{1},{\delta }_{1},{\delta }_{2}\right)={\lambda }_{1}(e)e$, $a{l}_{s}\left(e,{\alpha }_{2},{\delta }_{1},{\delta }_{2}\right)={\lambda }_{2}(e)e$, (21) can be written as:

22 $$\left\{\begin{array}{l}e={z}_{1}-{v}_{f}\\ {\mathrm{sz}}_{1}={z}_{2}-{\beta }_{1}{\lambda }_{1}(e)e+{b}_{0}u\\ {\mathrm{sz}}_{2}=-{\beta }_{2}{\lambda }_{2}(e)e\end{array}\right.$$

From (22), we get the following transfer function model:

23 $${z}_{1}=\frac{{\beta }_{1}{\lambda }_{1}\left(e\right){v}_{f}s+{\beta }_{2}{\lambda }_{2}\left(e\right){v}_{f}+{b}_{0}us}{{s}^{2}+{\beta }_{1}{\lambda }_{1}\left(e\right)s+{\beta }_{2}{\lambda }_{2}\left(e\right)}$$

24 $${z}_{2}=\frac{{\beta }_{2}{\lambda }_{2}\left(e\right)s{v}_{f}-{\beta }_{2}{\lambda }_{2}\left(e\right){b}_{0}u}{{s}^{2}+{\beta }_{1}{\lambda }_{1}\left(e\right)s+{\beta }_{2}{\lambda }_{2}\left(e\right)}$$

Parameter tuning of reference^16^, ${\beta }_{1}=3{w}_{0}$, ${\beta }_{2}=0.6{w}_{0}^{2}$, SESO has a good suppression effect on the system output, and the influence of u can be ignored for analysis.

25 $$\frac{{z}_{1}}{{v}_{f}}=\frac{3{w}_{0}s+0.6{w}_{0}^{2}}{{s}^{2}+3{w}_{0}s+0.6{w}_{0}^{2}}$$

**Figure 4:**
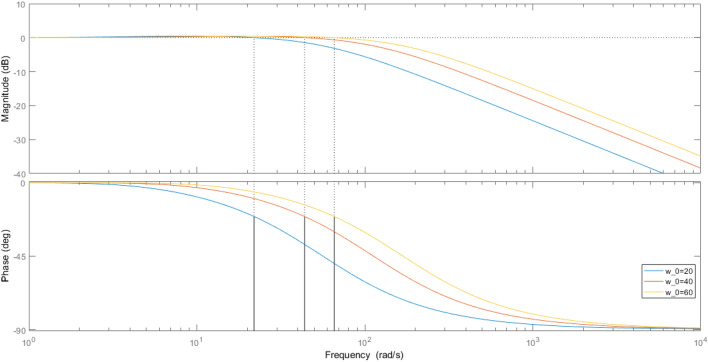
Bode plot of the transfer function.

The frequency domain characteristic analysis plot of the system can be obtained from the transfer function, and it can be seen from the Fig. 4, with the increase of ${w}_{0}$, the dynamic performance of SADRC is better. But in actual operation, the increase in ${w}_{0}$ also makes the motor control effect more ideal, too large ${w}_{0}$ will cause motor shaking and motor noise. Therefore, in the process of parameter adjustment, the ${w}_{0}$ should be gradually increased from a small value, and the ${w}_{0}$ when the motor is shaken is its critical value. that the smaller the ${\alpha }_{i}$, the stronger the nonlinearity of SADRC, but too small ${\alpha }_{i}$ will cause the motor to produce high-frequency oscillation, in this experiment, 0.25 and 0.5 were taken ${\alpha }_{1}$ and ${\alpha }_{2}$, respectively ${\delta }_{1}$ and ${\delta }_{2}$ determine the size of the nonlinear interval of SADRC, According to the system requirements, ${\delta }_{1}$ and ${\delta }_{2}$ are 0.05 and 1 respectively.

#### TD

**Figure 5:**
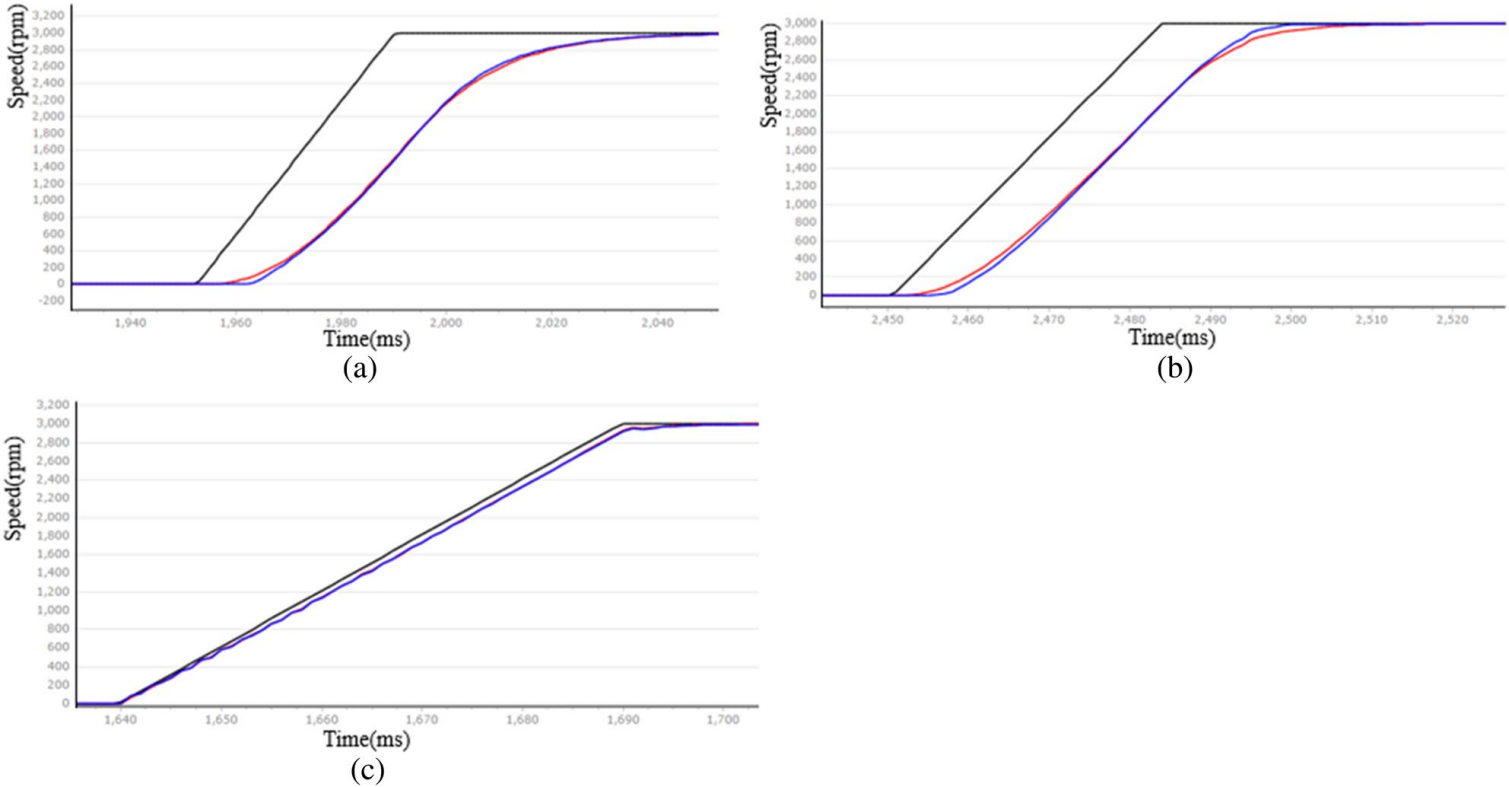
r velocity curve corresponding to different values (**a**) r = 5, (**b**) r = 10, (**c**) r = 200.

There are two adjustable parameters in this expression, step *h* and speed factor *r*. Step *h* is set to 0.01s, when the motor from the prohibition to the rated speed, the curve of different speed coefficients *r* is shown in Fig. 5, where the black line is the speed given, the red line is the speed given through the transition process of the speed output ${v}_{1}$, and the blue line is the speed feedback. The abscissa represents time, 4ms/scale, and the ordinate represents speed (the following coordinate diagrams are based on this standard).

This can be seen from Fig. 5, when *r* is 5 and 10, the rise time is 344ms and 192ms (sampled every 4ms), respectively, and when *r* increases to 200, the ${v}_{1}$ almost coincides with the given speed. Therefore, with the increase of r, the motor can reach the rated speed faster, but too large *r* will make the transition process invalid. In this experiment, *r* = 200 and *h* = 0.01 were taken.

#### SSEF

Introduce $fa{l}_{s}\left(e,{\alpha }_{i},{\delta }_{1},{\delta }_{2}\right)$ into the SEF, named SSEF. The state error feedback control rate is shown in Eq. (20), directly using proportional control, the pending parameters are: ${k}_{p}$, ${\alpha }_{1}{\prime}$, ${\delta }_{1}{\prime}$, ${\delta }_{2}{\prime}$, ${b}_{0}$ where ${\alpha }_{1}{\prime}$, ${\delta }_{1}{\prime}$, ${\delta }_{2}{\prime}$ are similar to the three parameters in SESO, and only need to be fine-tuned. In general, ${\delta }_{1}{\prime}$ should be within the interval [0.01, 0.1]. Therefore, in this experiment ${\alpha }_{1}{\prime}$, ${\delta }_{1}{\prime}$ and ${\delta }_{2}{\prime}$ are taken as 0.5, 0.1, and 1, respectively. ${b}_{0}$ determines the robustness of the system, the larger the ${b}_{0}$, the more stable the system, but the control effect is relatively poor. ${k}_{p}$ is similar to PD-controlled ${k}_{1}$, and the ${b}_{0}$ and ${k}_{p}$ of this experiment are taken as 20 and 380, respectively.

## Experimental results and analysis

### Experimental platform

**Figure 6:**
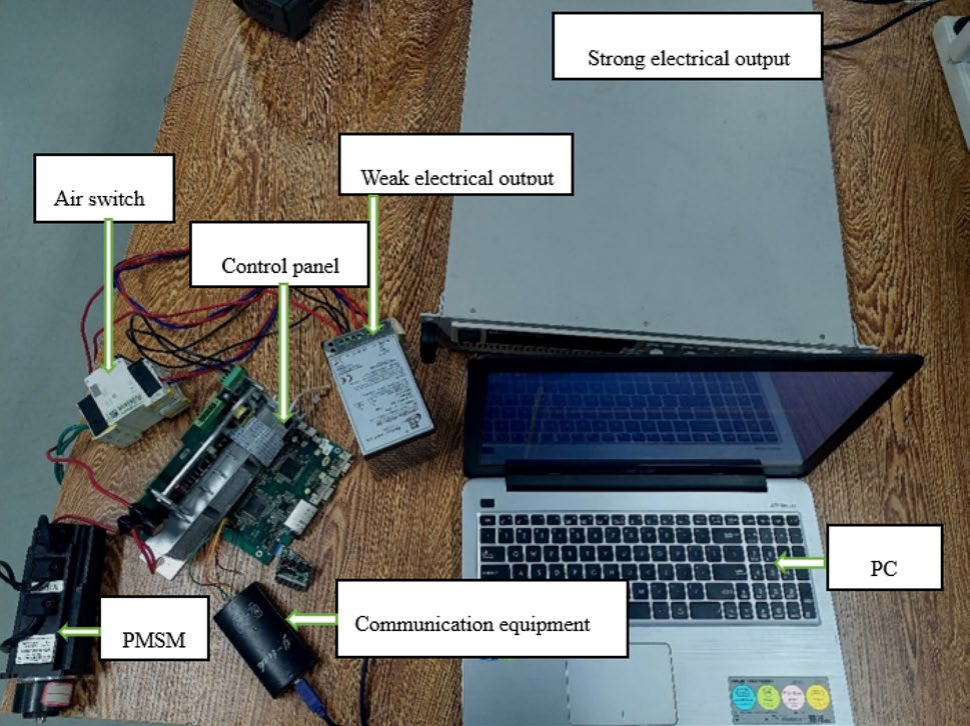
Experimental platform.

In order to evaluate the performance of SADRC, this paper conducts experiments on the following platform and compares the experimental data with that of LADRC and NLADRC. In this paper, the selected experimental motor is a 400W surface-mounted permanent magnet synchronous motor with a holding gate line. In both the step experiment and the steady-state experiment, the motor is in the under-load state with a load size of 1 kg, as shown in Fig. 6. Since position information is not required for the experiment, the speed-current double closed-loop mode is used. The absolute encoder monitors and provides real-time feedback on the motor speed. The surface-mount permanent magnet synchronous motor is a hidden-pole motor with a ${i}_{d}=0$ control strategy that ensures a linear relationship between the Q-axis current and the output torque. Table 1 provides a comprehensive list of parameters for PMSM.

**Table 1:** List of PMSM parameters.

Power (*P*)	400 W
Inertia ($J$)	0.315 × 10^−4^ kg m^2^
D-axis inductance (${L}_{d}$)	5.553 mH
Q-axis inductance ($${L}_{q})$$	5.553 mH
Number of pole pairs $({P}_{n}$)	5
Rated speed ($n$)	3000 rpm
Rated current ($I$)	2.9 A
Back electromotive-force constant	18.25 V/Krpm
Resistance	1.544 Ω

**Figure 7:**
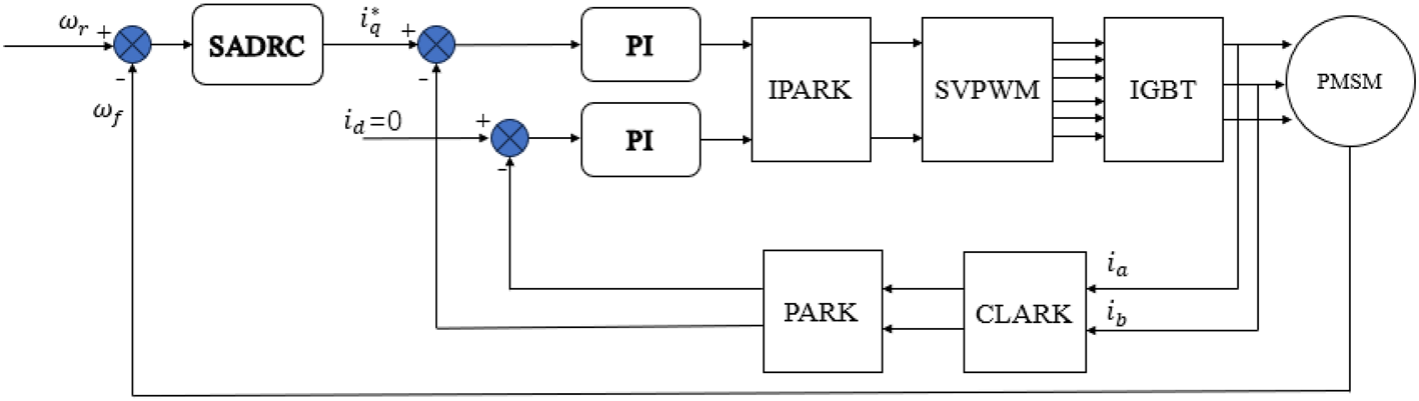
Servo control block diagram based on SADRC.

In this experiment, SADRC is applied to a servo-controlled speed loop, while the current loop is controlled by PI controller and the motor rotation is controlled by Field-Oriented Control (FOC). Closed-loop control is achieved by acquiring current information through the use of a sampling resistor. Finally, the experiment uses a PWM carrier frequency of 5kHz, and the specific control block diagram is shown in Fig. 7.

The rated speed of the motor is 3000 rpm, and the high current module can provide a voltage of 310V. The MCU adopts the STM32F407 chip, which has a maximum frequency of 168MHz. This frequency is sufficient to meet the control frequency requirements of the LADRC, NLADRC, and SADRC. The air switch is responsible for circuit protection, while the upper computer is responsible for algorithm parameter adjustment, data collection, and data collation.

### Step experiments

**Figure 8:**
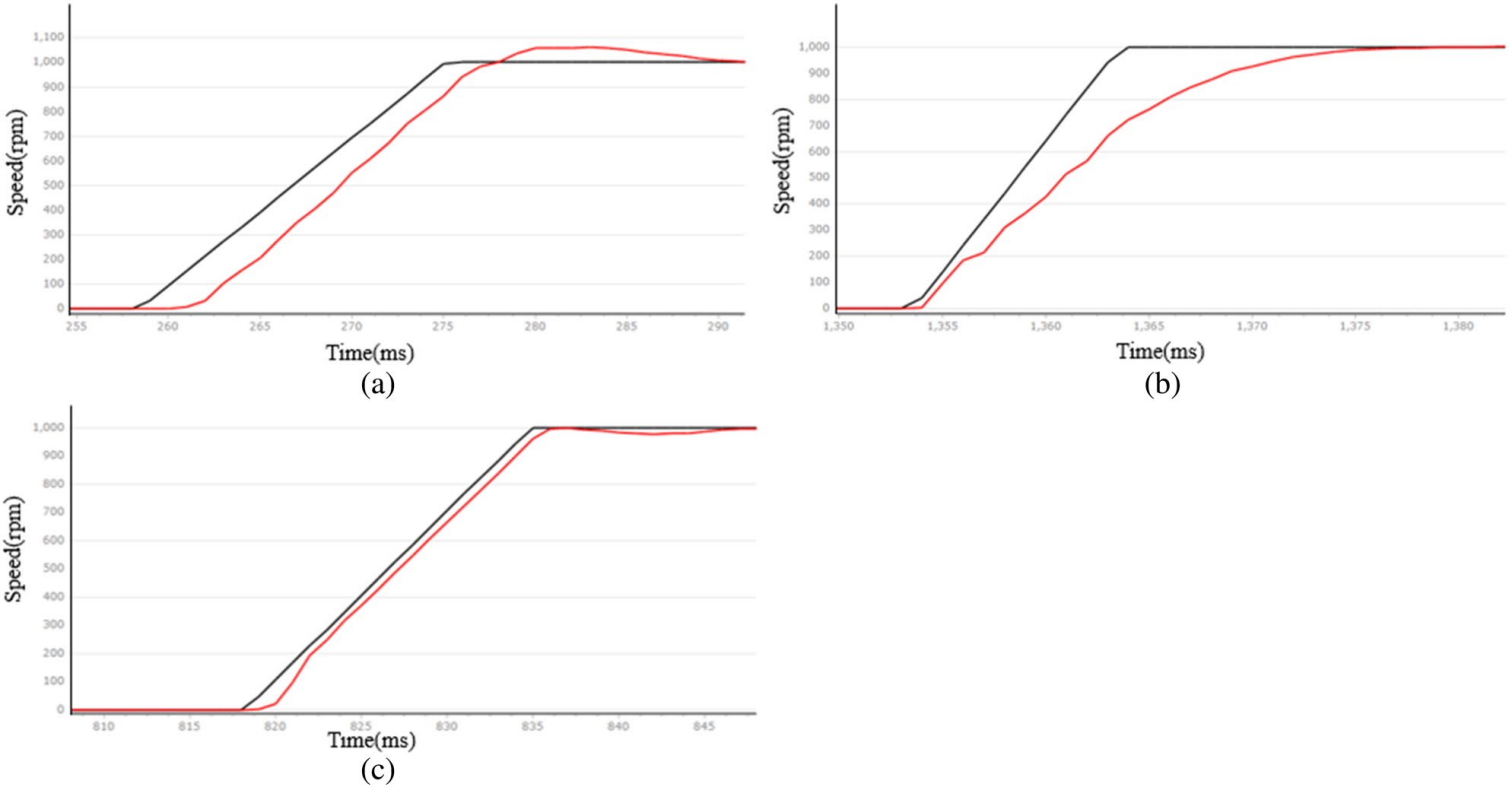
PMSM velocity rise curve at 1000 rpm given speed (**a**) LADRC, (**b**) NLADRC, (**c**) SADRC.

**Figure 9:**
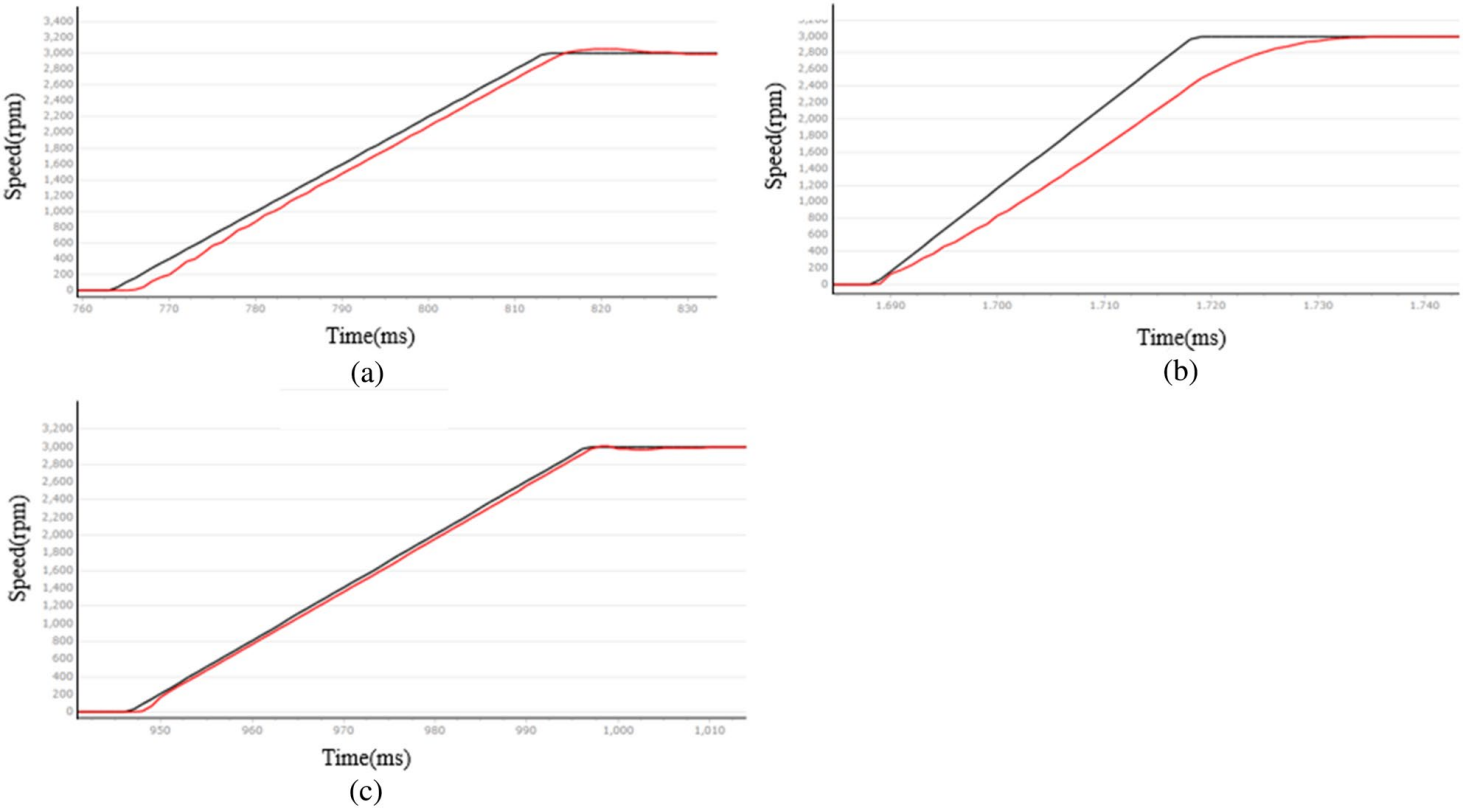
PMSM velocity rise curve at 3000 rpm given speed (**a**) LADRC, (**b**) NLADRC, (**c**) SADRC.

**Figure 10:**
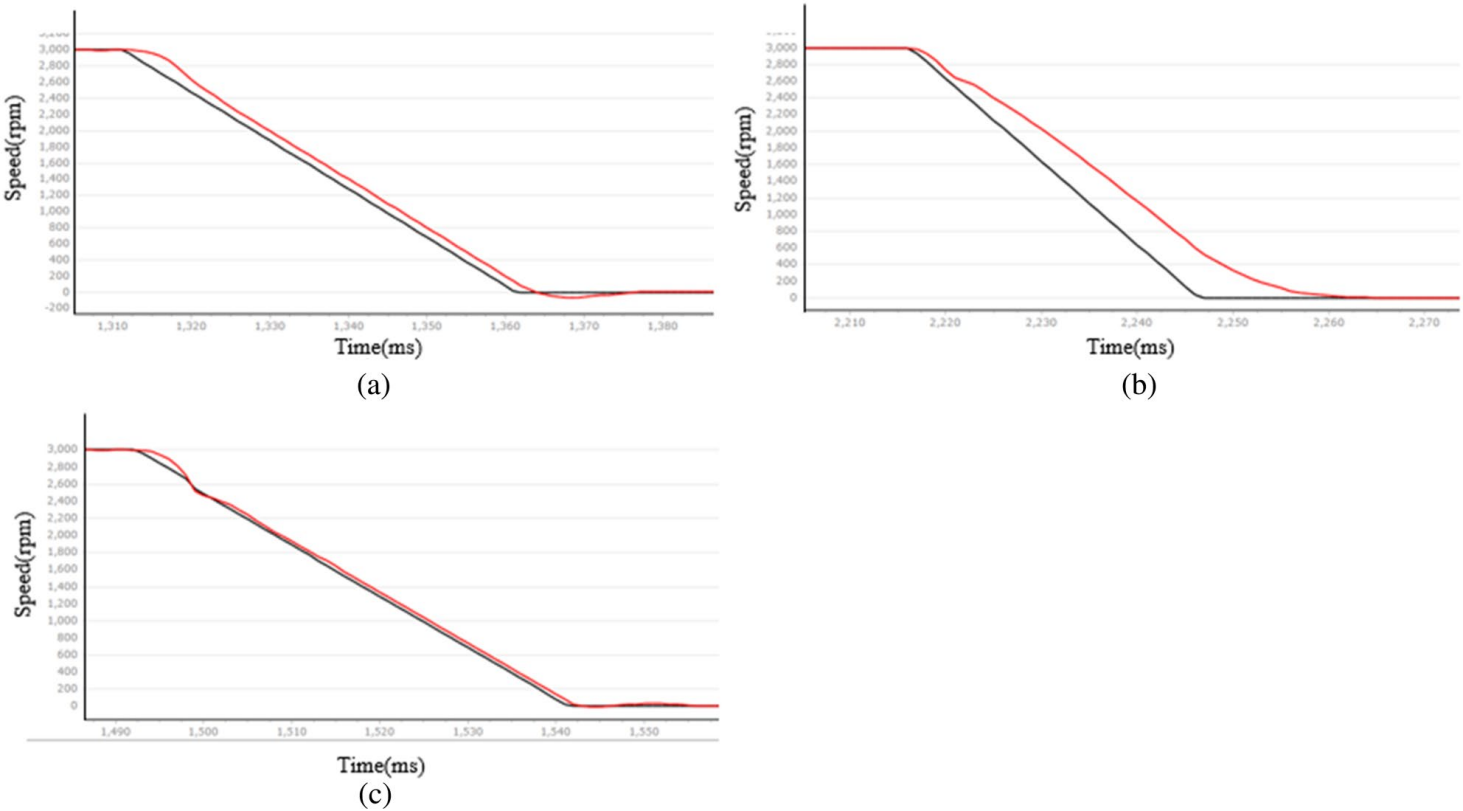
PMSM velocity decline curve at a given speed of 3000 rpm (**a**) LADRC, (**b**) NLADRC, (**c**) SADRC.

The essence of the industrial robotic arm's action lies in the rotation of the PMSM. The speed curve of the PMSM is analyzed at two given speeds: 1000 rpm and 3000 rpm. The black line represents the given speed curve, while the red line represents the speed feedback curve. This is shown in Figs. 8 and Fig. 9. Figure 10 shows the PMSM velocity decline phase curve. The horizontal axis of the following graph represents time, with a specific scale of 4 ms/1.

Therefore, the LADRC control has a positive impact on the speed rise and fall stages, but it experiences a delay during the initial start and results in significant overshoot. NLADRC deviates from the speed given curve in the rising and falling stages of the velocity feedback curve, and this deviation increases. This is due to the nonlinear function characteristics of NLADRC. As the error increases, the gain of NLADRC increases at a slower rate, which results in minimal overshoot in NLADRC speed feedback. SADRC has obvious advantages in terms of response time, overshoot, and adjustment time, and the specific data are shown in Table 2 (Supplementary Information).

**Table 2:** Comparison of PMSM start-stop performance.

Target rotational speed ${v}_{f}$ (rpm)	Control algorithms	Overshoot $\sigma$ (rpm)	Adjust the time ${t}_{s}$ (ms)
1000	LADRC	58	220
	NLADRC	4	112
	SADRC	2	72
3000	LADRC	62	344
	NLADRC	5	216
	SADRC	5	192

### Steady-state performance

**Figure 11:**
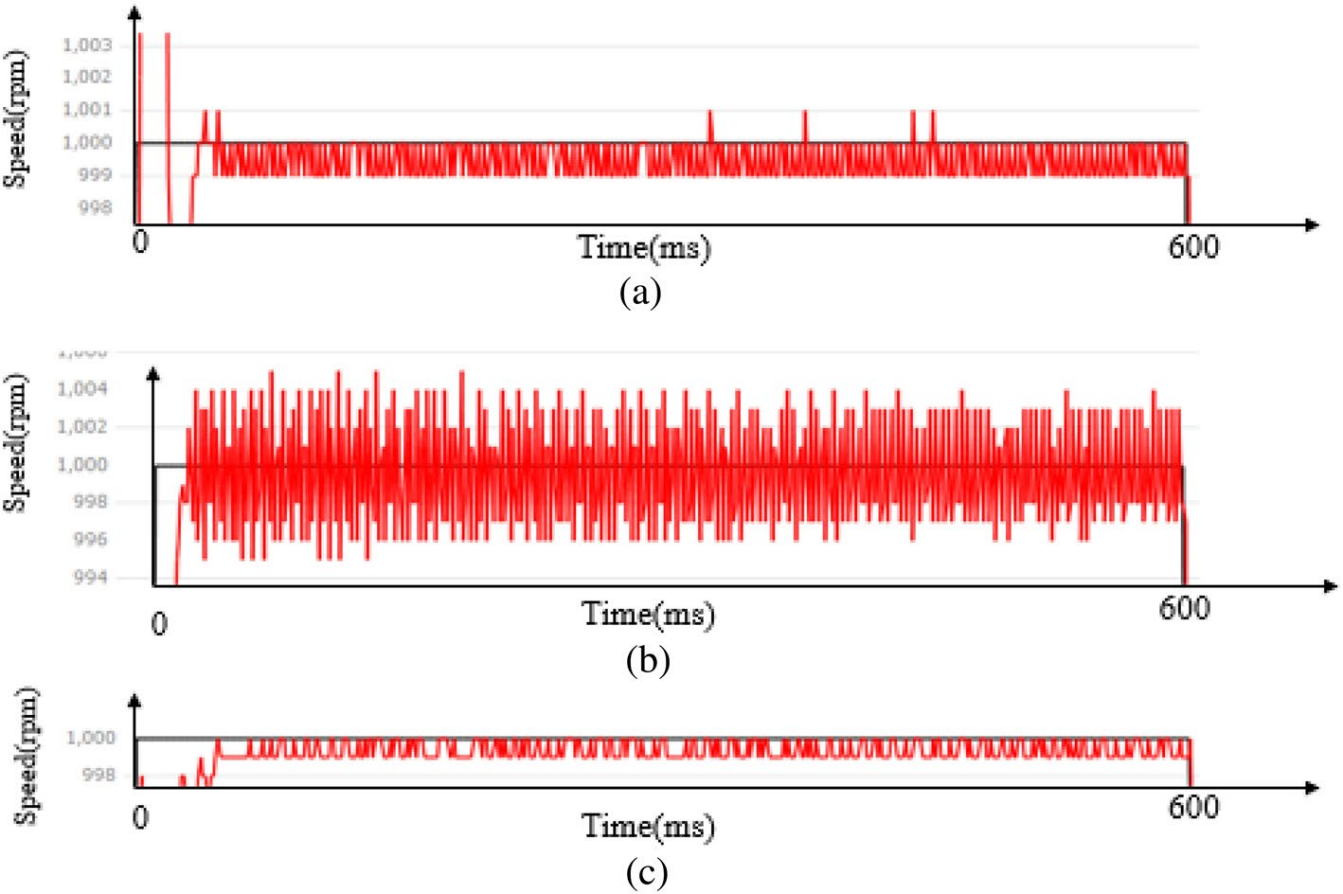
Steady-state curve of PMSM at a given speed of 1000 rpm (**a**) LADRC, (**b**) NLADRC, (**c**) SADRC.

The overall stability of the industrial robotic arm is determined by the steady-state performance of the motor. This paper compares the steady-state error of the speed curve of three control algorithms to reflect the steady-state performance, as shown in Fig. 11.

NLADRC is nonlinear and prone to high-frequency oscillations when the speed reaches a steady state. High-frequency oscillations can be attenuated by adjusting the corresponding parameters, but this may also lead to a deterioration in the control effect. Table 3 displays the average steady-state error for the three controllers.

**Table 3:** Comparison of PMSM steady-state errors.

	LADRC	NLADRC	SADRC
$\frac{\sum_{1}^{n}\left|e\right|}{n}$	0.72	1.89	0.66

### Load mutation experiments

**Figure 12:**
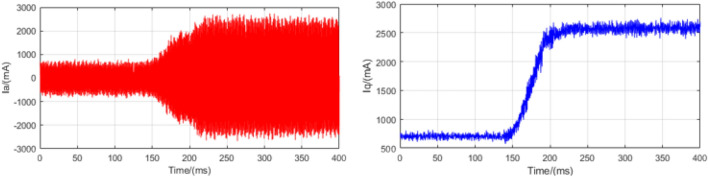
Load mutation experimental current plot based on LADRC.

**Figure 13:**
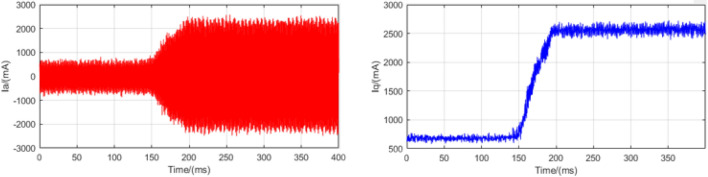
Load mutation experimental current plot based on NLADRC.

**Figure 14:**
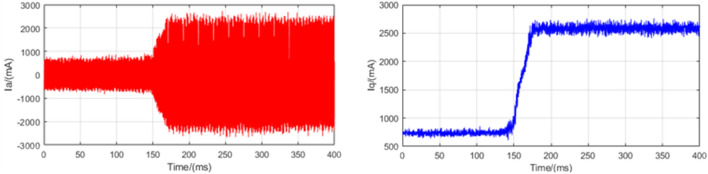
Load mutation experimental current plot based on SADRC.

In order to test the anti-disturbance ability of the control system, a load mutation experiment is designed. The experiment runs at a speed of 500 rpm and provides an instantaneous 1.5 N m moment to detect phase A current information. In order to enhance the accuracy of the system, we expanded the current information collected in this experiment. This is shown in Figs. 12, 13, and 14. Where red curve is the A-phase current and blue curve is the Q-axis current.

It can be seen from the experimental curve that during the load mutation of 1.5 N m, the anti-disturbance ability of LADRC is relatively weak. The specific data of current response time is shown in Table 4.

**Table 4:** Load mutation adjustment time.

	LADRC	NLADRC	SADRC
$\Delta t$(ms)	272	168	112

## Conclusion

In light of the advantages and disadvantages of LADRC and NLADRC in practical engineering, SADRC is designed to enhance the control effect of PMSM by incorporating the strengths of both LADRC and NLADRC. Due to the functional characteristics of $fa{l}_{s}\left(e,{\alpha }_{i},{\delta }_{1},{\delta }_{2}\right)$, SADRC can maintain good control effect when the error changes in a wide range. Through step experiments, steady-state experiments and load mutation experiments, the actual effects of the LADRC, NLADRC, and SADRC are compared, the experimental results verify the superiority of the SADRC.

### Supplementary Information

Supplementary Figures.

## Data Availability

The datasets generated or analyzed during this study are available from the corresponding author on reasonable request.
