# Supplementary material for: Research on permanent magnet synchronous motor algorithm based on linear nonlinear switching self-disturbance rejection control
Source: Sci Rep. 2023 Nov 16;13:20133. doi: 10.1038/s41598-023-46881-8 (PMC10656513; doi:10.1038/s41598-023-46881-8)
Supplement: Supplementary file 1 — Supplementary Figures. [file 41598_2023_46881_MOESM1_ESM.docx]

Additional diagrams for step experiments in manuscripts are shown below.

**
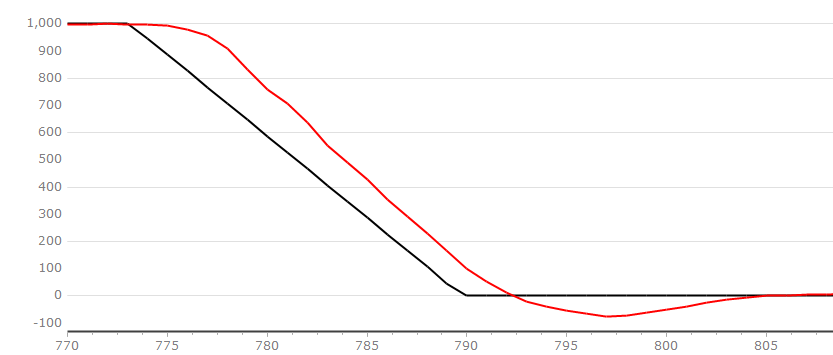
**


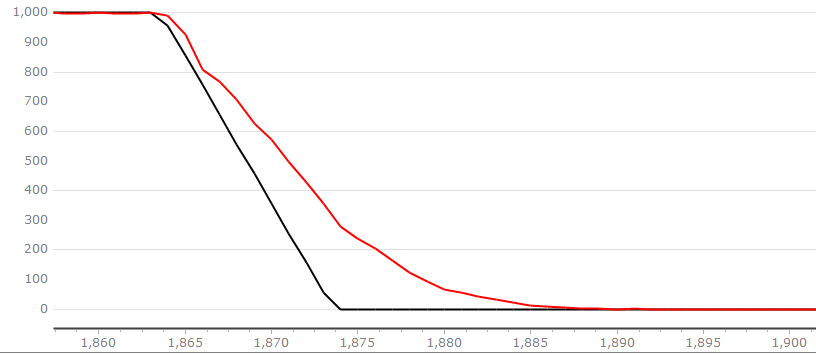


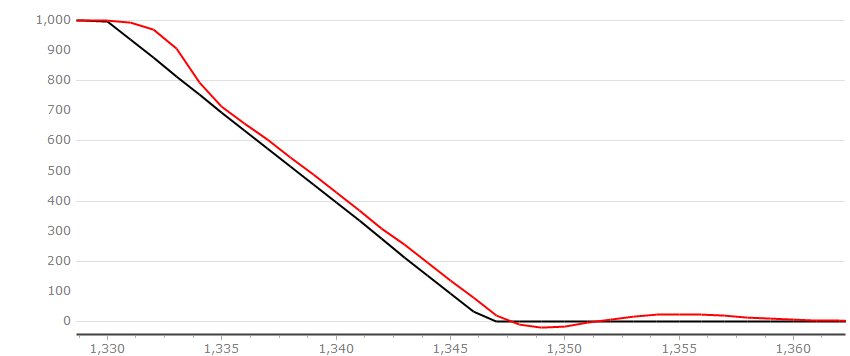
(a) (b)

(c)

Sl Figure 1. PMSM velocity decline curve at 1000 rpm given speed (a)LADRC (b)NLADRC (c)SADRC


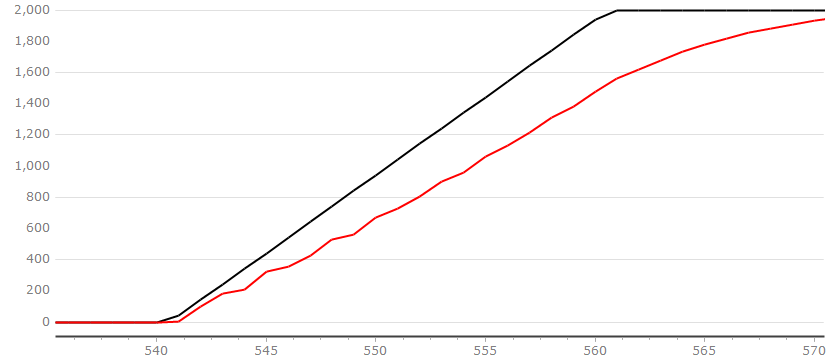

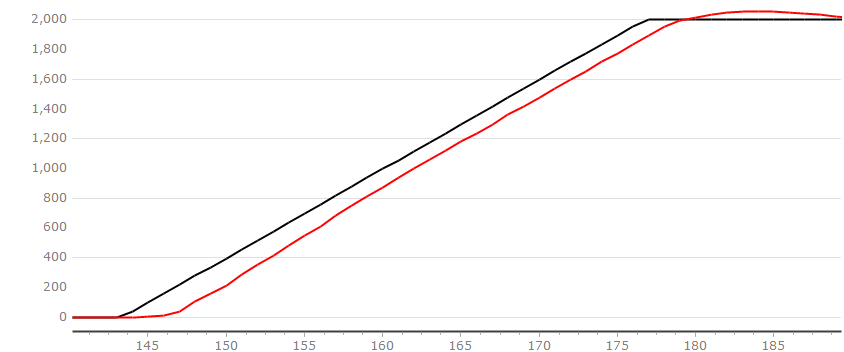


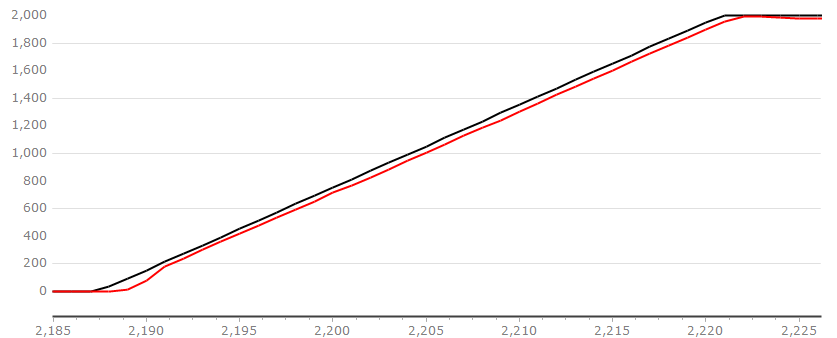
(a) (b)

(c)

Sl Figure 2. PMSM velocity rise curve at 2000 rpm given speed (a)LADRC (b)NLADRC (c)SADRC


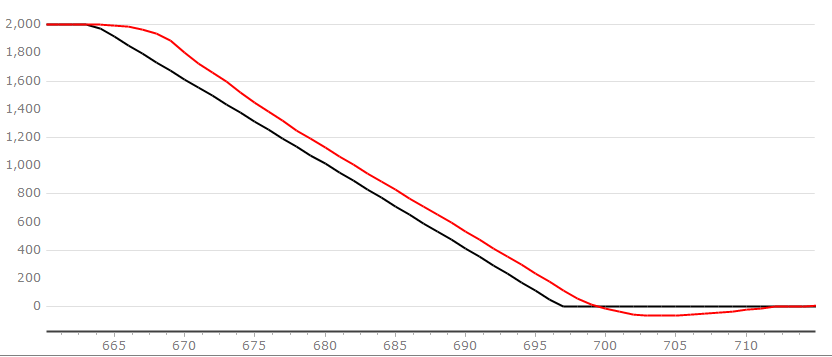

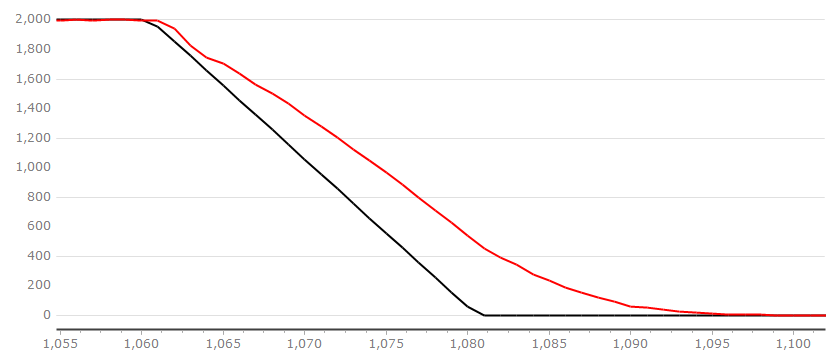


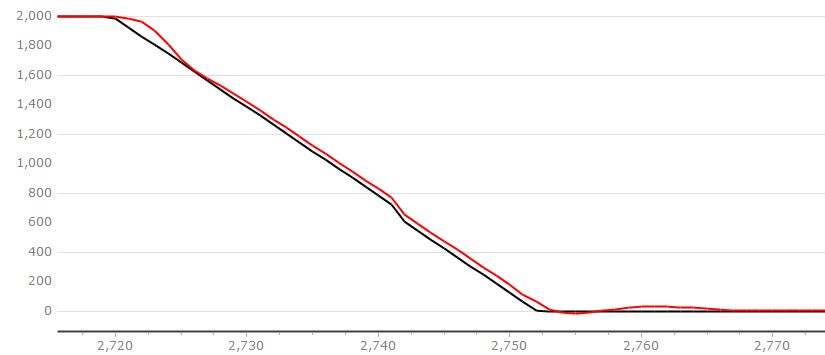
(a) (b)

(c)

Sl Figure 3. PMSM velocity decline curve at given speed of 2000 rpm (a)LADRC (b)NLADRC (c)SADRC
